# Long non-coding RNA *TUG1* regulates multiple glycolytic enzymes in hepatocellular carcinoma cells by sponging microRNA-122-5p

**DOI:** 10.7555/JBR.39.20250056

**Published:** 2025-07-15

**Authors:** Thammachanok Boonto, Chinnatam Phetkong, Chaiyaboot Ariyachet

**Affiliations:** 1 Department of Biochemistry, Faculty of Medicine, Chulalongkorn University, Bangkok 10330, Thailand; 2 Center of Excellence in Hepatitis and Liver Cancer, Faculty of Medicine, Chulalongkorn University, Bangkok 10330, Thailand; 3 Medical Biochemistry Program, Department of Biochemistry, Faculty of Medicine, Chulalongkorn University, Bangkok 10330, Thailand; 4 Medical Science Program, Faculty of Medicine, Chulalongkorn University, Bangkok 10330, Thailand

**Keywords:** hepatocellular carcinoma, *TUG1*, long-noncoding RNA, microRNA, glycolysis

## Abstract

Hepatocellular carcinoma (HCC) remains the third leading cause of cancer-related deaths worldwide; however, its therapeutic options are limited. Understanding the molecular mechanisms of HCC could provide insight into new therapies. Emerging studies indicate the important role of long-noncoding RNAs (lncRNAs) in the pathogenesis of HCC. The expression of the well-studied lncRNA taurine upregulated gene 1 ( *TUG1*) is upregulated in HCC tissues, but its transcriptomic effects in HCC cells remain unexplored. We established *TUG1*-knockdown and control HCC cells for RNA-seq experiments. KEGG analysis revealed glycolysis as the top enriched pathway upon *TUG1* silencing. Accordingly, *TUG1*-depleted HCC cells showed impairments in glucose uptake, ATP synthesis, and lactate production. Clinical HCC tissue data revealed positive gene expression correlations between *TUG1* and several glycolysis-related genes. To identify a molecular function of *TUG1* in glycolysis, we explored the competing endogenous model and used bioinformatic tools to find the five microRNAs (miRNAs) that had the most binding sites for *TUG1*. Among these miRNAs, miR-122-5p exhibited an inverse correlation in gene expression with most *TUG1*-regulated glycolysis genes, including *PKM*, *ALDOA*, *ENO2*, and *PFKM*. Dual-luciferase assays demonstrated the direct interaction between *TUG1* and miR-122-5p and between miR-122-5p and the 3ʹ untranslated regions of both *PKM* and *ALDOA*. We further showed that inhibition of miR-122-5p alleviated the suppression of glycolysis induced by *TUG1* depletion. Together, our RNA-seq analysis of *TUG1*-depleted HCC cells, combined with clinical data, reveals a critical role of *TUG1* in regulating glycolysis and provides new insight into its oncogenic function in HCC.

## Introduction

Hepatocellular carcinoma (HCC) is the most prevalent form of primary liver cancer, accounting for 80% of liver cancer diagnoses, and is the third leading cause of cancer-related deaths worldwide ^[ 1] ^. Progression of HCC is a multistep process that involves biological processes, including cellular metabolism, signaling pathways, cell cycle regulation, and epigenetic regulation, which are altered by the accumulation of genetic and epigenetic mutations, especially in patients with risk factors such as viral hepatitis, chronic alcohol use, smoking, diabetes, and metabolic-associated fatty liver disease ^[ 2] ^. Although mortality rates for HCC patients have decreased over the past decade, the therapeutic options for HCC remain unsatisfactory due to late diagnosis and limited treatment strategies for late-stage HCC ^[ 3] ^. Additionally, global incidence and mortality rates for HCC are expected to rise by 50% by 2040 ^[ 4] ^. Thus, a detailed understanding of HCC biology is essential for developing more effective diagnostic markers and therapeutic approaches.

Early HCC-related studies have highlighted the role of protein-coding genes in tumorigenesis. However, only approximately 2% of the human genome encodes protein-coding genes, while approximately 98% of the genome is transcribed into non-coding RNAs ^[ 5] ^. Among the various molecular factors, long non-coding RNAs (lncRNAs)—a class of non-coding RNAs longer than 200 nucleotides—have gained attention as crucial regulators in the development of cancers, including HCC ^[ 6] ^. Through their interactions with other biomolecules ( *e.g.*, DNA, RNA, and proteins), lncRNAs exhibit diverse regulatory functions at multiple levels of gene expression ^[ 7] ^. Based on numerous studies, aberrant expression of many lncRNAs has been implicated in several types of cancer and is directly linked to the oncogenic phenotype and the transformation of normal cells into tumor cells ^[ 8] ^.

Taurine upregulated gene 1 ( *TUG1*), one of the early identified lncRNAs that act as a tumor-promoting factor, plays significant roles in HCC development and progression ^[ 9] ^. Consistent with *in vitro* studies, *TUG1* expression is significantly increased in HCC tumor tissues, and patients with high expression of *TUG1* have a poor prognosis ^[ 10] ^. Numerous studies have suggested that *TUG1* primarily exerts its function as a competitive endogenous RNA to target miRNAs, thereby hindering their regulatory functions ^[ 11] ^. By sequestering regulatory miRNAs, *TUG1* can enhance a wide array of biological processes in HCC, including cell proliferation, migration, invasion, metabolic activity, and immune suppression, thus promoting the progression of HCC ^[ 12] ^. However, our understanding of the mechanistic function of *TUG1* in HCC remains incomplete. In this work, we performed a transcriptomic analysis of *TUG1*-depleted HCC cells and combined it with clinical data to elucidate the novel mechanism of oncogenic *TUG1* in HCC.

## Materials and methods

### Cell culture

HepG2 and SNU-449 cell lines were obtained from the American Type Culture Collection (Cat. #HB-8065 and CRL-2234, respectively; ATCC, Manassas, VA, USA). The JHH-4 cell line was obtained from the Japanese Collection of Research Bioresources (JCRB) Cell Bank (Cat. #JCRB0435, Osaka, Japan). HepG2 cells were maintained in low-glucose Dulbecco's Modified Eagle Medium (DMEM; Gibco, Detroit, MI, USA), supplemented with 10% fetal bovine serum (Cat. #SV3016003, Cytiva, Marlborough, MA, USA) and 1× antibiotic-antimycotic (Cat. #1TFS-1CC-15240062, Gibco), in a humidified atmosphere of 5% CO _2_ at 37 ℃. The culture medium and conditions for JHH-4 and SNU-449 cells were similar to those of HepG2, except replacing Eagle's minimal essential medium (EMEM; Cat. #1TFS-1CC-11095080, Gibco) for JHH-4 and Roswell Park Memorial Institute medium (RPMI; Cat. #1TFS-1CC-11875093, Gibco) for SNU-449 as the basal medium.

### Genetic perturbation of HCC cells

To inhibit the expression of *TUG1*, we used a lentivirus-mediated short hairpin (shRNA) vector that allowed temporal expression of shRNA upon doxycycline (DOX) treatment ^[ 13] ^. Two shRNAs targeting *TUG1* transcripts were designed using the GPP Web Portal (Broad Institute, Cambridge, MA, USA). Annealed oligonucleotides of *TUG1*-specific and control shRNAs were cloned into a lentiviral vector, Tet-pLKO-Puro (AddGene Plasmid #21915), and validated for successful insertion by Sanger sequencing. Lentivirus was produced in HEK293FT cells (Cat. #R70007, Thermo Fisher Scientific, Carlsbad, CA, USA) and used to establish DOX-inducible shRNA-control (sh-Con) or shRNA- *TUG1* (sh- *TUG1*) cell lines as previously described ^[ 13] ^. Most of the experiments used sh1- *TUG1* HCC lines, which demonstrated more than 50% *TUG1* knockdown efficiency after days of DOX treatment. To suppress the expression of microRNA-122-5p (miR-122-5p), control and miR-122-5p-specific antagomiRs were designed and cloned into Tet-pLKO-Puro and used to establish transgenic HCC cell lines with DOX-inducible expression of antagomiRs. All primers and sequences used for the shRNA and antagomiR cloning are shown in ***Supplementary Table 1*** (available online).

### Analysis of mRNA and miRNA expression

RNA extraction, reverse transcription, and real-time reverse transcription-PCR (qRT-PCR) analysis of mRNA and miRNA were performed as previously described ^[ 13] ^. Briefly, mRNA and miRNA were extracted from HCC cells using the GenUP Total RNA Kit and GenUP Micro RNA Kit, respectively, according to the manufacturer's protocols (Cat. #BR0700903 and BR0701903, respectively; Biotechrabbit, Berlin, Germany). A total of 500 ng of RNA per 10 μL reaction was reverse-transcribed into cDNA using iScript Reverse Transcription Supermix (Cat. #1708840, Bio-Rad, Hercules, CA, USA). qRT-PCR analysis was performed using a QuantStudio 5 Real-Time PCR System (Thermo Fisher Scientific) using 4× CAPITAL qPCR Probe Master Mix (Cat. #BR0501402, Biotechrabbit). The 2 ^−ΔΔCt^ method was used to analyze gene expression levels and normalize them to *RPL19* and *U6* for mRNA and miRNA, respectively. GAPDH primers were provided in the RevertAid First Strand cDNA Synthesis Kit (Cat. #K1622, Thermo Fisher Scientific). All primers used for qRT-PCR are shown in ***Supplementary Table 1***.

### RNA-sequencing (RNA-seq) analysis and gene set enrichment analysis (GSEA)

Total RNA was obtained from control and *TUG1* knockdown HepG2 cells for RNA-seq. Sequencing was conducted in three technical replicates. Transcriptomic analysis was performed by Biomarker Technologies (BMKGENE, Hong Kong, China). In brief, the purity and integrity of RNA samples were examined using the Agilent Bioanalyzer 2100 system (Agilent, Santa Clara, CA, USA). Sequencing libraries were prepared using the NEBNext Ultra RNA Library Prep Kit for Illumina (New England Biolabs, Ipswich, MA, USA). Sequencing was performed on the NovaSeq X platform (Illumina, San Diego, CA, USA). Differentially expressed genes (DEGs) between the control and *TUG1*-knockdown cells were identified using the criteria of |fold change (FC)| > 1.5 and *P* < 0.05 (adjusted for false discovery rate using the Benjamini–Hochberg method). To further investigate expression profiles upon *TUG1* depletion, ranked gene list analysis was performed using the GSEA software version 4.3.2. For this analysis, three gene set databases were used, including Hallmark (h.all.v2023.2), WikiPathways (c2.cp.wikipathways.v2023.2), and Kyoto Encyclopedia of Genes and Genomes (KEGG) MEDICUS (c2.cp.kegg_medicus.v2023.2). Gene sets with an FDR *q*-value < 0.05 were considered significantly upregulated and downregulated. The RNA-seq data sets generated in the current study are available in the National Center for Biotechnology Information Gene Expression Omnibus repository under the accession code GSE273253.

### Bioinformatic analysis of clinical data and RNA interaction

Gene and miRNA expression levels of HCC patients were derived from TCGA using the TCGAbiolinks package. In brief, gene expression data were queried with the following parameters: project = TCGA-LIHC, data.category = Transcriptome Profiling, and data.type = Gene Expression Quantification. For miRNA expression, parameters used were project = TCGA-LIHC, experimental.strategy = miRNA-Seq, data.category = Transcriptome Profiling, data.type = Isoform Expression Quantification. For the survival analysis, the expression profiles of HCC patients were derived from the OncoLnc database in the LIHC dataset, queried by gene ID or miRNA accession (MIMAT0000421: miR-122-5p). Correlation analysis was performed using GraphPad Prism software version 10.3.0 (GraphPad) and derived from the GEPIA and starBase v2.0. To identify novel *TUG1*-targeting miRNAs, the multiMiR package with predicted datasets was employed on a list of *TUG1*-associated glycolysis genes. To identify the microRNA response elements (MREs) of miR-122-5p, the RNA22 v2 microRNA prediction tool was used to obtain the putative binding sites of *TUG1*.

### Proliferation and glycolysis-related assays

Cell proliferation was assessed by the MTT assay, which measures metabolic activity to quantify the number of living cells. Briefly, approximately 5 × 10 ^4^ cells/well were seeded in a 96-well plate and grown for four days. Each day, cells were treated with the MTT labeling reagent (0.5 mg/mL; Cat. #M6494, Invitrogen, Carlsbad, CA, USA) for 2 h. After the end of incubation, the purple formazan crystals were solubilized in DMSO, and the absorbance was measured using the BioTek Synergy HTX microplate reader (BioSPX, Abcoude, Belgium) at 570 nm.

Glucose uptake into HCC cells was measured by the Glucose Uptake-Glo Assay (Cat. #J1341, Promega, Madison, WI, USA). Briefly, approximately 2 × 10 ^5^ cells/well were seeded in a 96-well plate and treated with 1 mmol/L of 2-deoxyglucose in PBS for 10 min. Cells were then lysed to stop all cellular activities, and the uptake of 2-deoxyglucose was measured by a luminescent signal in a detection reagent buffer containing glucose-6-phosphate dehydrogenase (G6PDH), nicotinamide adenine dinucleotide phosphate (NADP ^+^), reductase, proluciferin substrate, and luciferase catalysis.

Levels of adenosine triphosphate (ATP) production were measured by the CellTiter-Glo 2.0 Cell Viability Assay (Cat. #G9241, Promega). Briefly, approximately 2 × 10 ^5^ cells/well were seeded in a 96-well plate and lysed to release ATP, which generates a luminescent signal proportional to the amount of ATP present *via* catalysis of luciferase.

Lactate production was assessed by the Lactate-Glo Assay (Cat. #J5021, Promega). Briefly, approximately 2 × 10 ^5^ cells/well were seeded in a 96-well plate in a basal medium containing 10% dialyzed fetal bovine serum for 2 h. The culture medium was then harvested and quantified for the amount of lactate by a luminescent signal in a lactate reaction reagent containing lactate dehydrogenase, nicotinamide adenine dinucleotide (NAD ^+^), reductase, proluciferin substrate, and luciferase.

### Dual luciferase reporter assays

Wild-type and mutant sequences of *TUG1* MREs and the 3ʹ untranslated regions (3ʹ UTRs) of *PKM* and *ALDOA* transcripts were cloned into the SacⅠ/XhoⅠ-cut pmirGLO plasmid (Cat. #E1330, Promega). The miR-122-5p sequence was cloned into the BamHⅠ/HindⅢ-cut pSilencer plasmid (Cat. #AM7210, Thermo Fisher Scientific). The primers used for cloning are listed in ***Supplemental Table 1***. The interaction between miR-122-5p and its predicted binding sites (MREs and 3ʹ UTRs) was tested using the Dual-Luciferase Reporter Assay System (Cat. #1910, Promega) according to the manufacturer's instructions. Briefly, approximately 2 × 10 ^5^ HEK293FT cells/well were seeded in a 96-well plate, and 300 ng of insert-containing pmirGLO and pSilencer plasmids were transfected into the cells using the standard calcium phosphate transfection protocol. After 24 h of transfection, the cells were lysed, sequentially incubated with firefly and Renilla luciferase substrates, and measured for luminescence using the BioTek Synergy HTX microplate reader. Firefly luciferase levels were normalized to *Renilla* luciferase levels to demonstrate miRNA binding efficiency.

### Western blotting analysis

Proteins from HCC cells were prepared in radioimmunoprecipitation (RIPA) lysis buffer containing the complete protease inhibitor cocktail (Cat. #04693132001, Roche, Basel, Switzerland), separated by 12% SDS-PAGE, and transferred to a nitrocellulose membrane using a Trans-Blot SD Semi-Dry Transfer Cell (Bio-Rad). Membranes were then incubated in the BlockPRO 1 Min Protein-Free Blocking Buffer (Cat. #BM01-500, Visual Protein, Taipei, Taiwan, China), and probed overnight with the following primary antibodies: PKM (1∶2000; Cat. #A0268, ABclonal, Woburn, MA, USA), ALDOA (1∶2000; Cat. #A1142, ABclonal), and β-actin (1∶2000; Cat. #sc-47778, Santa Cruz Biotechnology, Dallas, TX, USA). HRP-conjugated goat anti-mouse IgG and goat anti-rabbit IgG (1∶5000, Cat. #7076 and Cat. #7074, respectively; Cell Signaling Technology, Danvers, MA, USA) were used as secondary antibodies. Blots were imaged using a UVP ChemStudio instrument (Analytik Jena, Beverly, MA, USA). The quantification of protein levels was normalized to β-actin. Uncropped blots are shown in ***Supplementary Fig. 1*** (available online).

### Statistical analysis

Data were expressed as mean ± standard deviation and statistically analyzed using GraphPad Prism software version 10.3.0. The differences between groups were compared using an unpaired Student's *t-*test or one-way ANOVA. Three or more technical replicates from at least two independent experiments were used for the determination of significant differences for each HCC cell line. Results were validated in three independent HCC cell lines for functional experiments. *P* < 0.05 was considered statistically significant.

## Results

### Transcriptomic analysis of *TUG1*-depleted HCC cells

**Figure 1:**
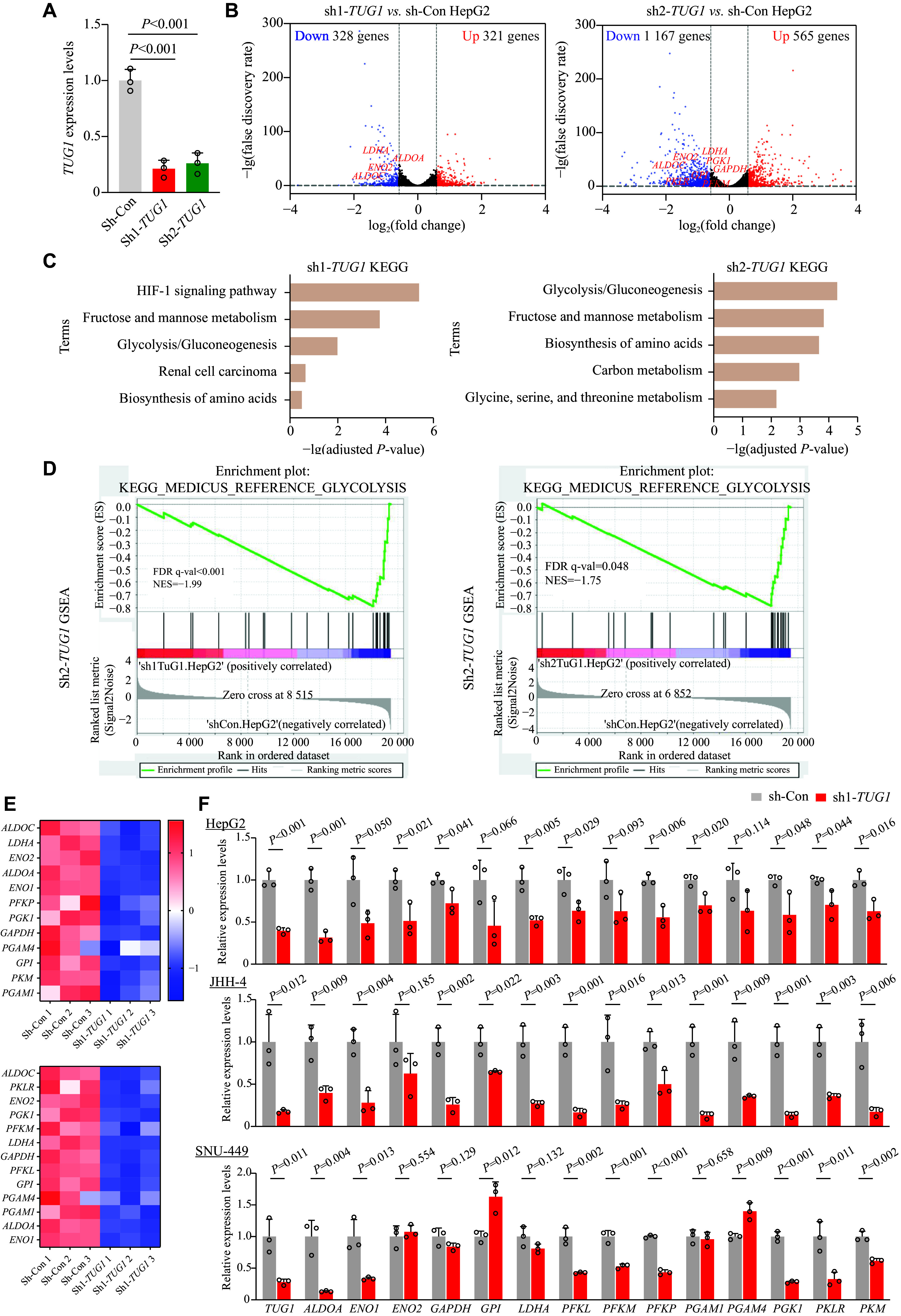
Transcriptomic analysis of *TUG1*-knockdown HCC cells. A: *TUG1* expression upon shRNA-mediated depletion determined by real-time reverse transcription-PCR (qRT-PCR). B: Volcano plots depicting the log _2_(fold change [FC]) (x-axis) of gene expression in *TUG1*-knockdown (sh1- and sh2- *TUG1*) and control (sh-Con) HepG2 cells, as well as the statistical significance −lg(false discovery rate) (y-axis). Differentially expressed genes (DEGs) were identified using the criteria of |FC| > 1.5 and *P* < 0.05, where upregulated and downregulated DEGs are represented as red and blue dots, respectively, with highlights for DEGs in the glycolysis pathway. C: Kyoto Encyclopedia of Genes and Genomes (KEGG) analysis of downregulated DEGs in *TUG1*-knockdown HepG2 cells. D: Gene Set Enrichment Analysis (GSEA)-based KEGG enrichment plots of representative gene sets from the glycolysis pathway. E: Expression levels of representative glycolysis-related genes in control and *TUG1*-knockdown HepG2 cells from RNA-seq analysis. F: Expression of representative glycolysis-related genes upon *TUG1* depletion with sh1- *TUG1*, compared with control, determined by qRT-PCR in three HCC cell lines (HepG2, JHH-4, and SNU-449). Data are presented as mean ± standard deviation and normalized to those of sh-Con HCC cells (set as 1.0). Three technical replicates were performed for each HCC line. Indicated *P*-values were determined by Student's *t*-test.

Although *TUG1* is a well-known oncogenic lncRNA, its influence on the global transcriptome remains unknown. To explore the biological roles of *TUG1* in HCC, we performed a transcriptomic analysis of HCC cells after *TUG1* depletion. We silenced *TUG1* expression with two independent shRNAs and established two stable *TUG1* knockdown lines in HepG2 cells (sh1- *TUG1* and sh2- *TUG1*), along with control cells (sh-Con). The two shRNAs targeting *TUG1* exhibited comparable knockdown efficiency ( ***Fig. 1A***). We obtained libraries of isolated mRNAs from control and *TUG1*-knockdown HepG2 cells and performed RNA-seq for transcriptomic analysis to reveal the biological processes influenced by *TUG1*. In sh1- *TUG1* HepG2 cells, we observed upregulation of 321 DEGs and downregulation of 328 DEGs, while in sh2- *TUG1* HepG2 cells, we observed upregulation of 565 DEGs and downregulation of 1167 DEGs, compared with the control ( ***Fig. 1B*** and ***Supplementary File 1*** [available online]). We performed KEGG and GO analyses of downregulated DEGs, and the results showed that *TUG1* depletion significantly affected the glycolysis/gluconeogenesis pathway in both knockdown cell lines ( ***Fig. 1C*** and ***Supplementary Fig. 2*** [available online]). We employed GSEA to further determine the role of *TUG1* in this biological pathway, and the results showed that glycolysis was among the top-enriched gene sets following *TUG1* knockdown ( ***Fig. 1D*** and ***Supplementary Table 2*** [available online]). In *TUG1*-knockdown HepG2 cells, various glycolysis-related genes were notably downregulated, including aldolase ( *e.g.*, *ALDOA* and *ALDOC*), enolase ( *e.g.*, *ENO1* and *ENO2*), phosphoglycerate mutase ( *e.g.*, *PGAM1* and *PGAM4*), phosphofructokinase ( *e.g.*, *PFKL*, *PFKM*, and *PFKP*), and pyruvate kinase ( *e.g.*, *PKLR* and *PKM*) ( ***Fig. 1E***). We further validated the downregulation of these glycolysis-related genes using qRT-PCR in three different HCC cell lines with *TUG1* knockdown ( ***Fig. 1F***). Together, these results suggest that *TUG1* positively regulates the expression of multiple genes in the glycolysis pathway in HCC cells.

Our RNA-seq data revealed differences in downregulated DEGs between the sh1- *TUG1* and sh2- *TUG1* groups. To further investigate these differences, we analyzed the overlapping downregulated DEGs. Using a stringent threshold of |FC| > 1.5 and *P* < 0.05, we identified 236 overlapping DEGs, but KEGG pathway enrichment analysis did not reveal significant downregulation of the glycolysis pathway ( ***Supplementary Fig. 3A*** and ***3B*** [available online]). However, when we relaxed the threshold to |FC| > 1.0 and *P* < 0.05, the overlap increased to 979 genes, and glycolysis emerged as a significantly downregulated pathway ( ***Supplementary Fig. 3C*** and ***3D*** [available online]). These results suggest that while individual genes may exhibit modest fold changes, the overall pathway-level effect of *TUG1* knockdown on glycolysis remains significant.

### *TUG1* knockdown impaired proliferation and glycolysis in HCC cells

**Figure 2:**
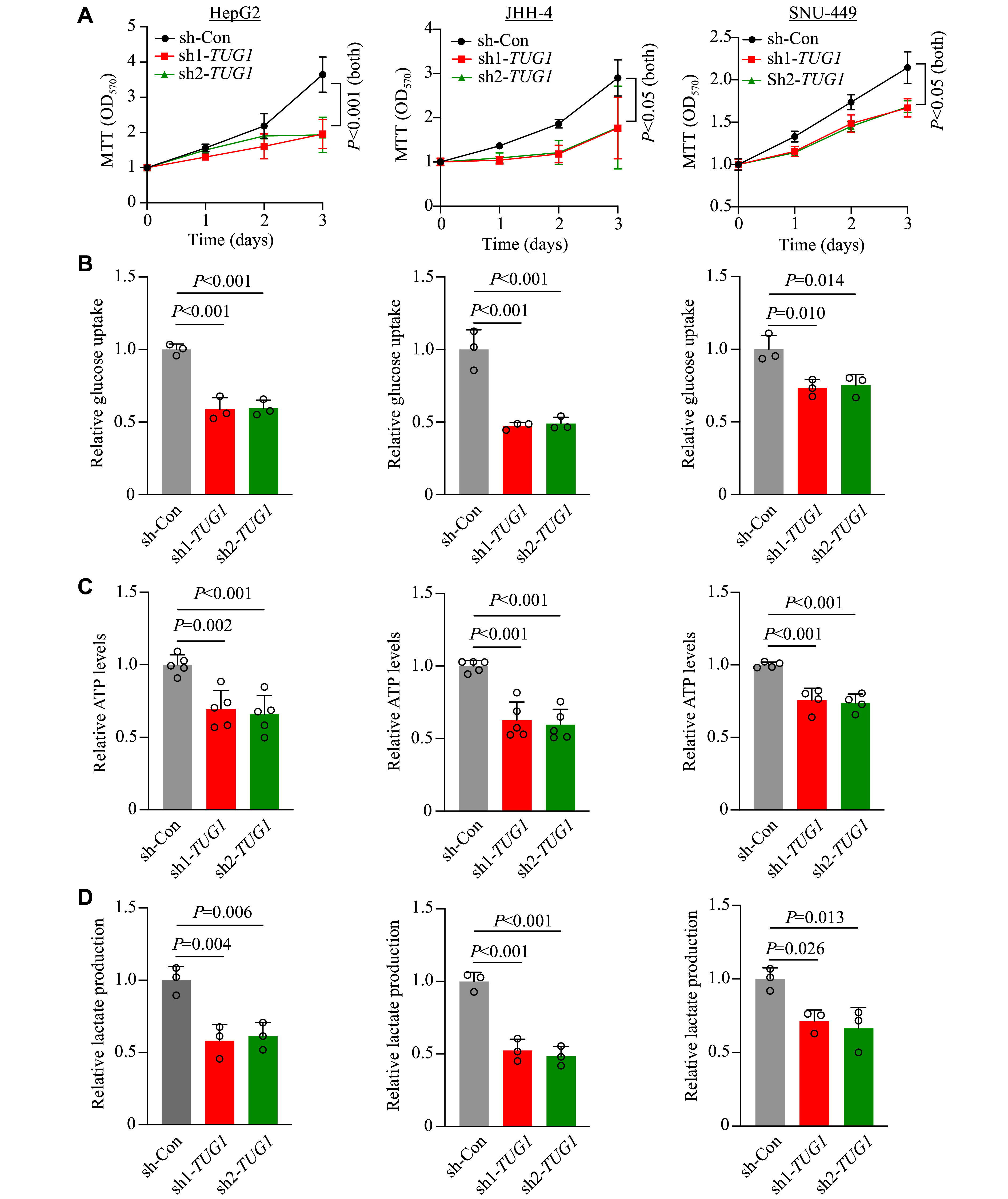
Knockdown of *TUG1* impaired proliferation and glycolytic activity in HCC cells. A: MTT proliferation assays on control and *TUG1*-knockdown HCC cells. B: Glucose uptake by control and *TUG1*-knockdown HCC cells. C: ATP production by control and *TUG1*-knockdown HCC cells. D: Lactate formation in control and *TUG1*-knockdown HCC cells. All assays were performed on three HCC cell lines (HepG2, JHH-4, and SNU-449). Data are presented as the mean ± standard deviation of *n* ≥ 3 replicates from at least two independent experiments from each cell line and normalized to those of sh-Con HCC cells (set as 1.0) for all experiments. One-way ANOVA with indicated *P*-values shown in the figure. The specific *P*-values for the MTT assay are as follows: for HepG2, sh-Con *vs*. sh1- *TUG1*and sh-Con *vs*. sh2- *TUG1*, *P* < 0.001; for JHH-4, sh-Con *vs.* sh1- *TUG1*, *P* = 0.014, and sh-Con *vs*. sh2- *TUG1*, *P* = 0.040; for SNU-449, sh-Con *vs.* sh1- *TUG1*, *P* = 0.018, and sh-Con *vs.* sh2- *TUG1*, *P* = 0.016. Abbreviation: MTT, 3-(4,5-dimethylthiazolyl-2)-2,5-diphenyltetrazolium bromide.

Our RNA-seq analysis suggested glycolysis as a top-affected pathway after *TUG1* depletion. Various types of cancer, including HCC, exhibit the 'Warburg effect', a phenomenon characterized by enhanced glycolytic activity, which perpetuates disease progression by supplying essential building blocks to sustain cancer's rapid growth ^[ 14] ^. We performed MTT assays to evaluate proliferation after *TUG1* knockdown, and found that the suppression of *TUG1* inhibited cell proliferation in three independent HCC cell lines ( ***Fig. 2A***). According to our RNA-seq analysis, we hypothesized that impaired glycolysis could be responsible for the growth defect. We then examined glycolytic activity, in which glucose is the initial substrate of the process, and its inhibition slows down glucose uptake *via* glucose transporters ^[ 14] ^. Indeed, we observed impaired glucose transport rates in *TUG1*-knockdown HCC cells, which aligned with our hypothesis ( ***Fig. 2B***). Cancer cells utilize glucose to generate ATP *via* enhanced glycolysis, and we observed a significant reduction in ATP levels in *TUG1*-depleted HCC cells, further supporting the impairment of glycolysis ( ***Fig. 2C***). Different kinds of cancer cells often overproduce lactate, the final product of aerobic glycolysis ^[ 14] ^. In line with the glucose transport and ATP production results, we also observed a reduction in lactate production from HCC cells upon *TUG1* knockdown, likely because of impairments in glucose uptake and glycolysis ( ***Fig. 2D***). Together, these results suggest that *TUG1* plays a crucial role in modulating the glycolysis pathway in HCC cells.

### Positive correlation between *TUG1* expression and glycolysis gene expression in HCC tissues

**Figure 3:**
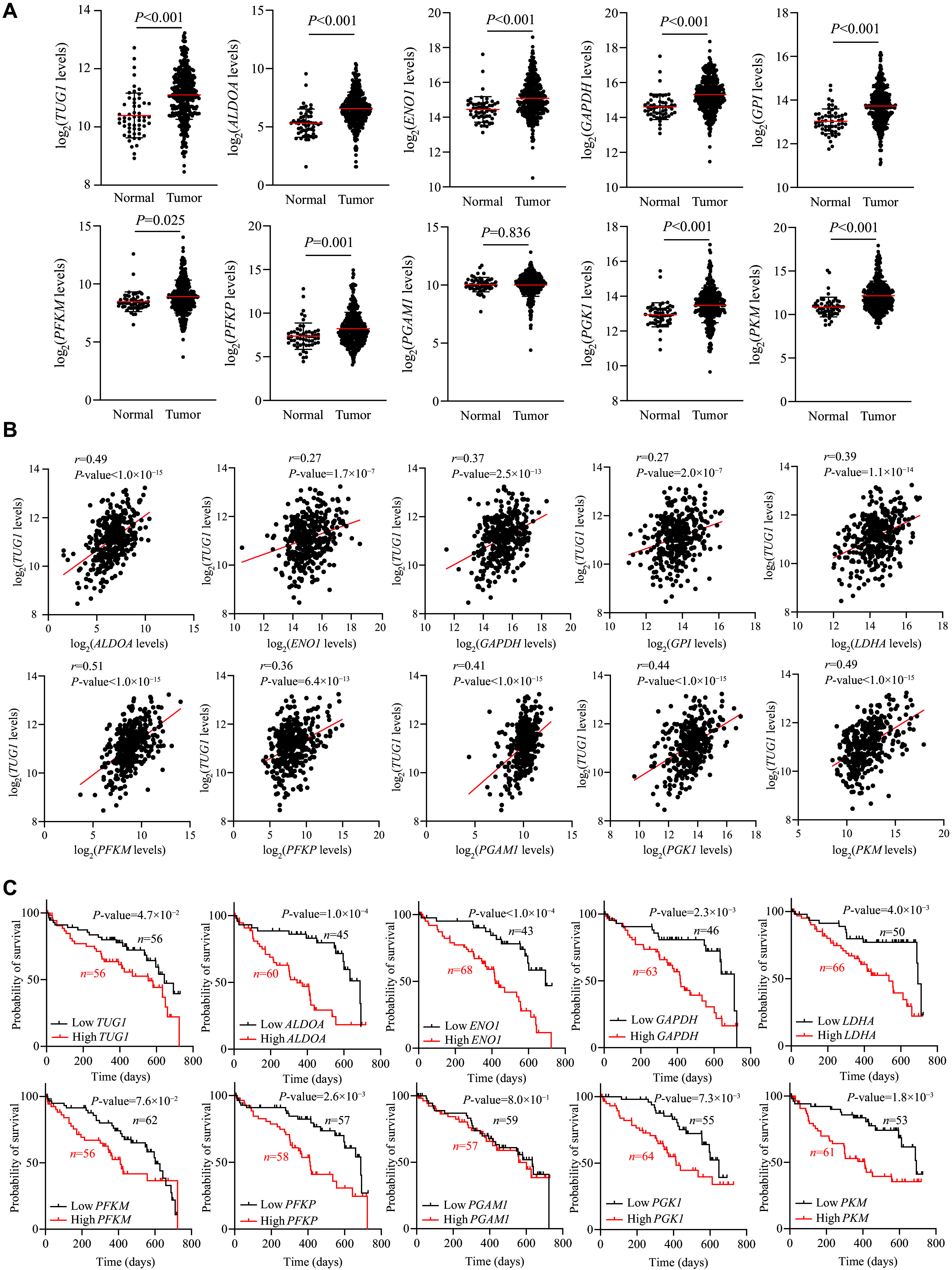
*TUG1* and glycolysis genes were upregulated, positively correlated, and associated with overall survival in HCC. A: Expression levels of *TUG1* and glycolysis genes in normal ( *n* = 54) and HCC tissues ( *n* = 368) from the TCGA database. Student's *t*-test with indicated *P*-values shown in the figure. B: Spearman correlation ( *r*) between expression levels of *TUG1* (y-axis) and glycolysis genes (x-axis) from healthy and HCC samples. C: Kaplan–Meier analysis of the correlation between *TUG1*/glycolysis genes and two-year overall survival in the TCGA cohort. Log-rank tests were used to determine the statistical significance ( *P* < 0.05).

Next, to determine the clinical relevance of the *TUG1*-associated glycolysis genes, we examined their expression levels in tumor versus normal tissues of HCC patients from the TCGA database. Indeed, the expression levels of *TUG1* and most of the glycolysis-related genes, except *PGAM1*, were significantly upregulated in tumor tissues, compared with adjacent normal tissues ( ***Fig. 3A***). We further determined the correlation between *TUG1* and glycolysis-related genes and found that the majority of glycolysis-related genes exhibited a positive correlation with *TUG1*, indicating that *TUG1* may exert regulatory effects on these genes ( ***Fig. 3B*** and ***Supplementary Table 3*** [available online]). Furthermore, we employed survival analysis on genes associated with *TUG1* to assess their influence on the prognostic outcomes of HCC patients, and found that HCC patients with higher expression of glycolysis genes experienced poorer prognoses ( ***Fig. 3C***). Taken together, these results suggest the tumorigenic role of *TUG1* in HCC and that its overexpression may enhance glycolysis, resulting in accelerated proliferation of HCC cells and contributing to the unfavorable prognosis of HCC patients.

### *TUG1* modulated activity of the antiglycolytic miR-122-5p

**Figure 4:**
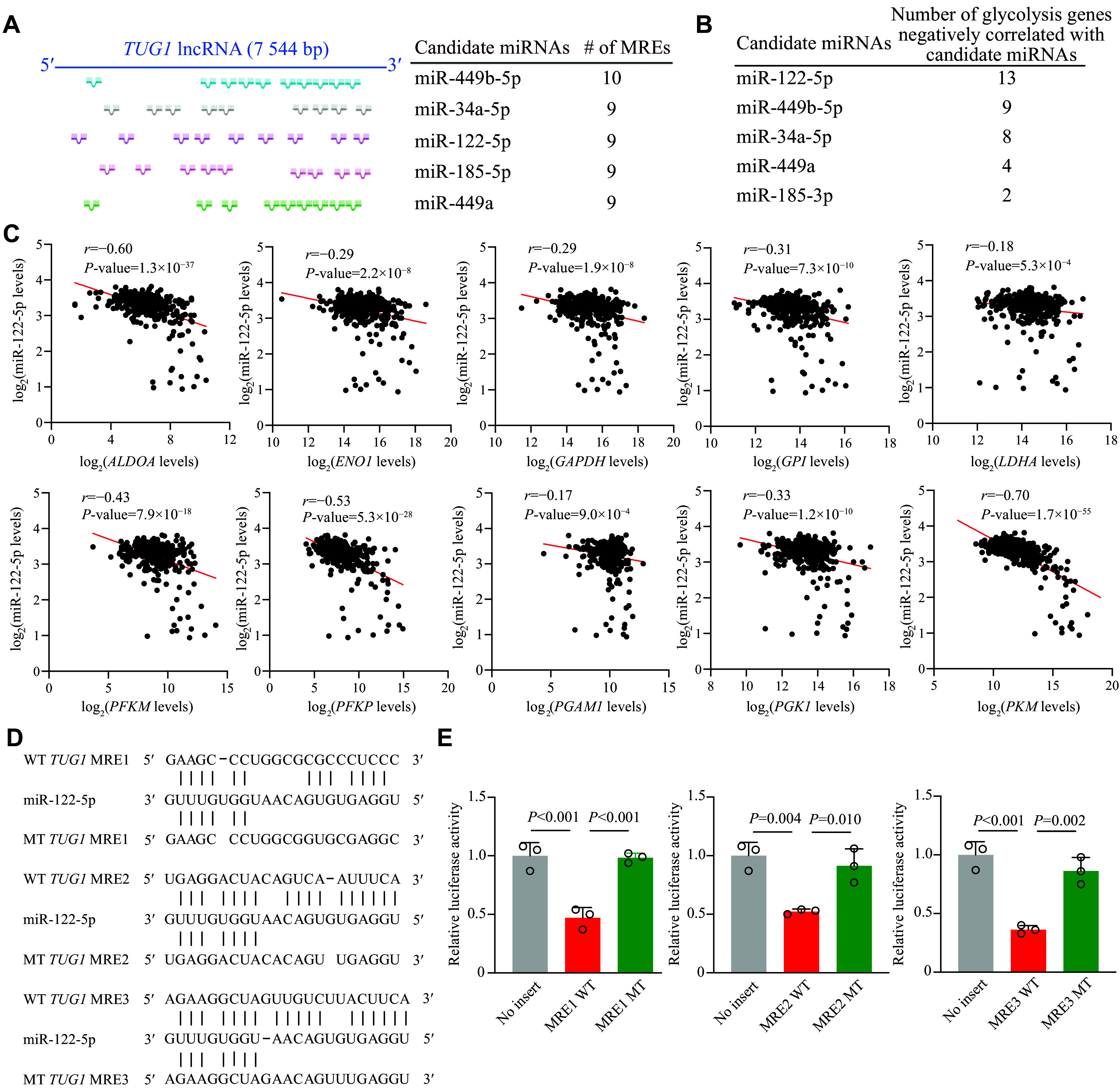
*TUG1* acted as a sponge for miR-122-5p. A: Schematic diagram depicting miRNAs with the top number of microRNA response elements (MREs) within the *TUG1* sequence as predicted by RNA22 v2 microRNA target detection. B: The number of *TUG1*-associated glycolysis genes that were negatively correlated with the expression of candidate miRNAs in normal and HCC tissues. C: Spearman correlation ( *r*) between expression levels of miR-122-5p (y-axis) and glycolysis genes (x-axis) in healthy and HCC samples. D: Hybridization patterns of wild-type (WT) and mutant (MT) *TUG1* MRE sequences and miR-122-5p. The top three MREs of candidate binding sites for miR-122-5p are shown (MRE1–3). E: Dual luciferase reporter assays were performed to validate the interaction of miR-122-5p with WT and MT *TUG1* MRE1–3 sequences. Data are presented as the mean ± standard deviation and normalized to those of the no-insert control (set as 1.0). *n* = 3 technical replicates. One-way ANOVA with indicated *P*-values shown in the figure.

One of the molecular mechanisms by which an lncRNA may influence biological pathways in cancer is through sequestering tumor-suppressor miRNAs. We hypothesized that *TUG1* could sponge miRNAs that negatively regulate the activity of glycolysis, thus enhancing the glycolytic activity of HCC. To identify novel target miRNAs for *TUG1*, we examined a list of candidate miRNAs with the highest interaction score with the *TUG1*-associated glycolysis genes ( ***Supplementary Table 4*** [available online]). Then, we computationally predicted the number of MREs of candidate miRNAs in *TUG1* to determine the likelihood of interaction between *TUG1* and candidate miRNAs. We obtained the top five candidates with the highest number of MREs, including miR-449b, miR-34a-5p, miR-122-5p, miR-185-3p, and miR-449a ( ***Fig. 4A*** and ***Supplementary Table 5*** [available online]). Subsequently, we investigated the inverse correlation between candidate miRNAs and glycolysis-related genes in the TCGA database. Our goal was to find miRNAs that could potentially modulate these genes with clinical relevance. Of the five candidates, miR-122-5p exhibited the most pronounced negative correlation with these genes, suggesting that this miRNA may serve as a regulator of glycolysis ( ***Fig. 4B*** and ***Supplementary Table 6*** [available online]). Additionally, *ALDOA* and *PKM* showed the strongest inverse correlation among the negatively correlated genes, reinforcing the likelihood that these genes could be targets of miR-122-5p ( ***Fig. 4C***). To verify the direct interaction between *TUG1* and miR-122-5p, we performed a dual-luciferase reporter assay focusing on the miR-122-5p MREs within *TUG1* ( ***Fig. 4D***). Indeed, the results showed that miR-122-5p significantly reduced luciferase activity in the *TUG1*-MRE wild-type reporter, while no such repression was observed with the *TUG1*-MRE mutant sequence ( ***Fig. 4E***). Overall, the results suggest that *TUG1* may sequester miR-122-5p, thereby stimulating glycolytic activity in HCC.

### miR-122-5p directly interacted with the 3ʹ UTR of both *ALDOA* and *PKM* transcripts

**Figure 5:**
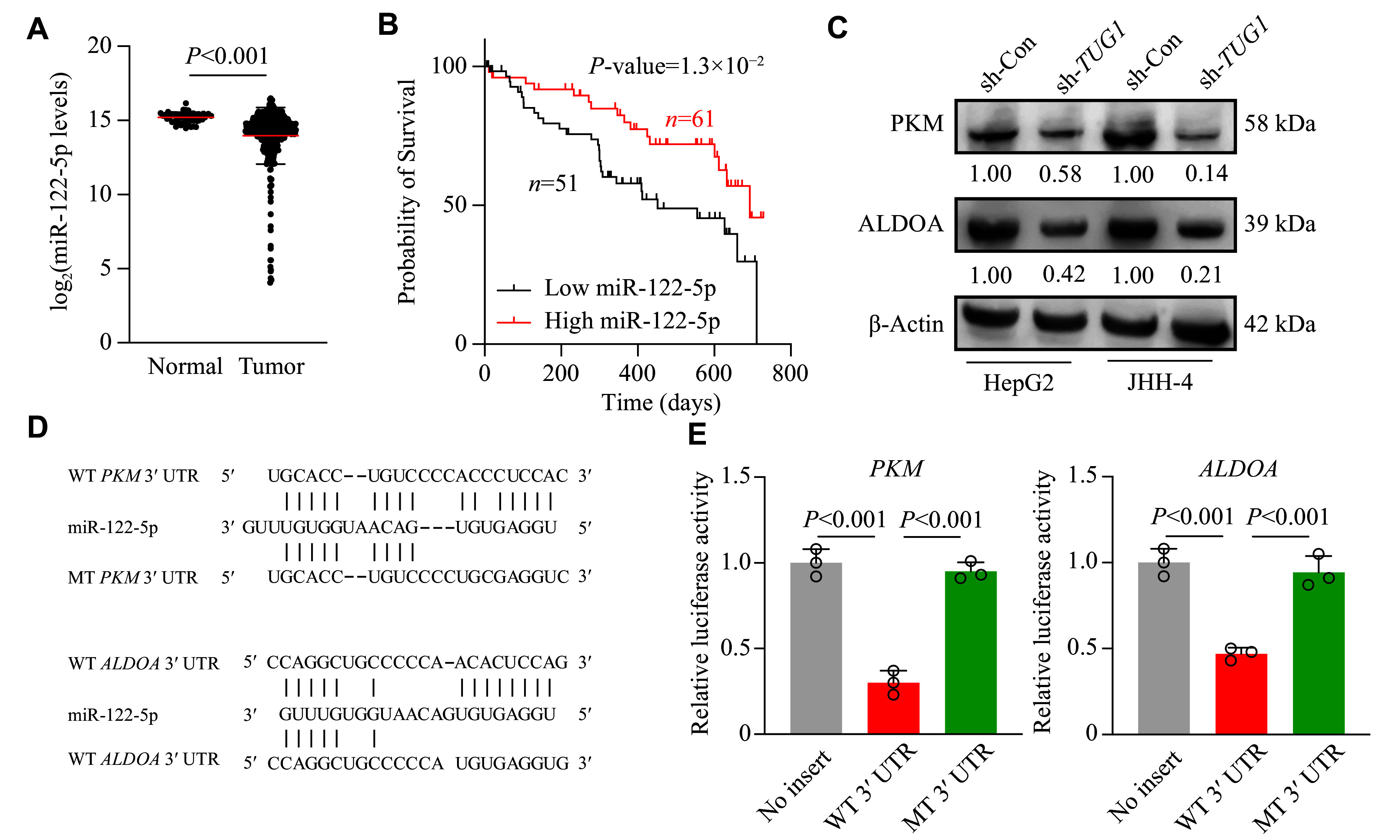
miR-122-5p directly regulated the expression of *PKM* and *ALDOA*. A: Expression levels of miR-122-5p in normal ( *n* = 54) and HCC tissues ( *n* = 368) from the TCGA database. Student's *t*-test with indicated *P*-values shown in the figure. B: Kaplan–Meier analysis of the correlation between miR-122-5p and two-year overall survival in the TCGA cohort. Log-rank tests were used to determine the statistical significance ( *P* < 0.05). C: Protein levels of PKM and ALDOA in control and *TUG1*-knockdown HCC cells were determined by Western blotting. Protein levels were normalized to β-actin for quantification. D: Hybridization pattern of wild-type (WT) and mutant (MT) *PKM* and *ALDOA* 3′ UTR sequences with miR-122-5p. E: Dual luciferase reporter assays were performed to assess the interaction between WT and MT *PKM* and *ALDOA* 3′ UTR sequences and miR-122-5p. Data are presented as the mean ± standard deviation and normalized to those of the no-insert control (set as 1.0). *n* = 3 technical replicates. One-way ANOVA with indicated *P*-values shown in the figure.

Our re-analysis of clinical data indicates that miR-122-5p may hinder the promotion of glycolysis in HCC ( ***Fig. 4C***). To support our findings, we analyzed the expression levels of miR-122-5p in HCC patients from the TCGA database and observed a significant downregulation in tumor tissues, compared with adjacent normal tissues, supporting its role as a tumor-suppressing miRNA ( ***Fig. 5A***). Moreover, survival analysis demonstrated that HCC patients with high miR-122-5p expression exhibited improved prognosis, compared with those with low miR-122-5p expression, suggesting a protective role of miR-122-5p in HCC ( ***Fig. 5B***). Among the *TUG1*-associated glycolysis genes, *ALDOA* and *PKM* exhibited the most substantial inverse correlation with miR-122-5p, implying that they may be regulated by miR-122-5p ( ***Fig. 4C*** and ***Supplementary Table 6*** [available online]). We performed Western blotting to explore the effect of *TUG1* on ALDOA and PKM at the protein level, and found that when *TUG1* was depleted, ALDOA and PKM protein levels decreased significantly in two separate HCC cell lines, consistent with their RNA levels ( ***Fig. 5C*** and ***Fig. 1F***). Next, we further demonstrated the direct interaction of miR-122-5p with the 3ʹ UTR of both *ALDOA* and *PKM* transcripts using the dual luciferase reporter gene assay ( ***Fig. 5D*** and ***Supplementary Table 7*** [available online]). Indeed, the results showed that miR-122-5p interacted directly with the 3ʹ UTR of both *PKM* and *ALDOA* mRNAs ( ***Fig. 5E***). Collectively, we identified *ALDOA* and *PKM* as the direct targets of miR-122-5p, and *TUG1* could enhance *ALDOA* and *PKM* expression to stimulate glycolysis by sequestering miR-122-5p.

### Reduced miR-122-5p levels alleviated the repression of glycolysis in *TUG1*-depleted cells

**Figure 6:**
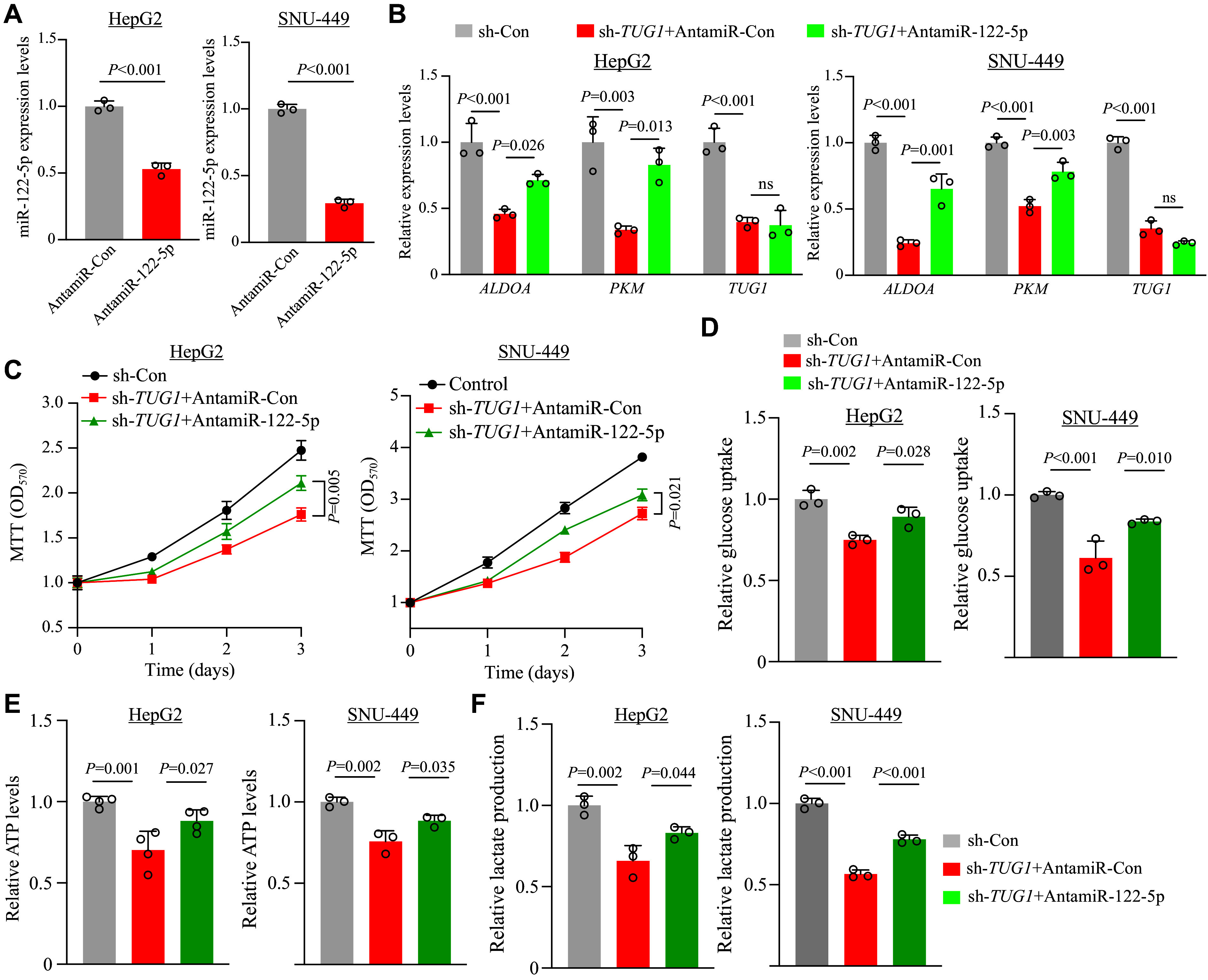
miR-122-5p was important for *TUG1*-mediated suppression of glycolysis in HCC cells. A: Real-time reverse transcription-PCR (qRT-PCR) analysis of miR-122-5p levels in HCC cells (HepG2 and SNU-449) transfected with control antagomiR (antamiR-Con) and miR-122-5p antagomiR (antamiR-122-5p). B: qRT-PCR analysis of *PKM*, *ALDOA*, and *TUG1* transcripts in *TUG1*-knockdown HCC cells co-transfected with antamiR-Con or antamiR-122-5p. C–F: Control and TUG1-knockdown HCC cells co-transfected with antamiR-Con or antamiR-122-5p were analyzed for proliferation (MTT assay, C), glucose uptake (D), ATP production (E), and lactate formation (F). Data are presented as the mean ± standard deviation of *n* ≥ 3 technical replicates from each cell line and normalized to those of sh-Con HCC cells (set as 1.0) for all experiments. One-way ANOVA with indicated *P*-values shown in the figure. Abbreviation: MTT, 3-(4,5-dimethylthiazolyl-2)-2,5-diphenyltetrazolium bromide; ns, not significant.

Our findings demonstrated that *TUG1* could promote both *ALDOA* and *PKM* expression by sponging the antiglycolytic miR-122-5p. To provide further evidence that miR-122-5p is crucial in modulating glycolysis in HCC through the *TUG1*/miR-122-5p/ *ALDOA*/ *PKM* axis, we introduced control antagomiR (antamiR-Con) or miR-122-5p antagomiR (antamiR-122-5p) into two independent *TUG1*-knockdown HCC cells. To begin with, we tested the efficiency of antamiR-122-5p, and the results showed that antamiR-122-5p effectively reduced the miR-122-5p levels ( ***Fig. 6A***). Then, we examined the expression of glycolysis genes and found that antamiR-122-5p partially enhanced the expression of *ALDOA* and *PKM* in *TUG1*-knockdown HCC cells ( ***Fig. 6B***). Since earlier observations showed that *TUG1* depletion in HCC cells caused impairment in cell proliferation, we then performed MTT assay and found that depletion of miR-122-5p partially restored proliferation after *TUG1* knockdown ( ***Fig. 6C***). Subsequently, we investigated whether knockdown of miR-122-5p could reverse the defective glycolysis in *TUG1*-knockdown HCC cells. We observed that glucose uptake levels were moderately enhanced in HCC cells with miR-122-5p inhibitors ( ***Fig. 6D***). In line with the restoration of the glucose uptake, ATP levels in *TUG1*-depleted cells were partially elevated upon knockdown of miR-122-5p ( ***Fig. 6E***). Additionally, increased lactate production was observed with the depletion of miR-122-5p in *TUG1*-knockdown HCC cells ( ***Fig. 6F***). Taken together, the restoration of cell proliferation and glycolytic activity upon miR-122-5p depletion highlights the tumorigenic function of *TUG1*, which sequesters the antiglycolytic miR-122-5p and upregulates *ALDOA* and *PKM* expression to promote glycolysis in HCC.

## Discussion

**Figure 7:**
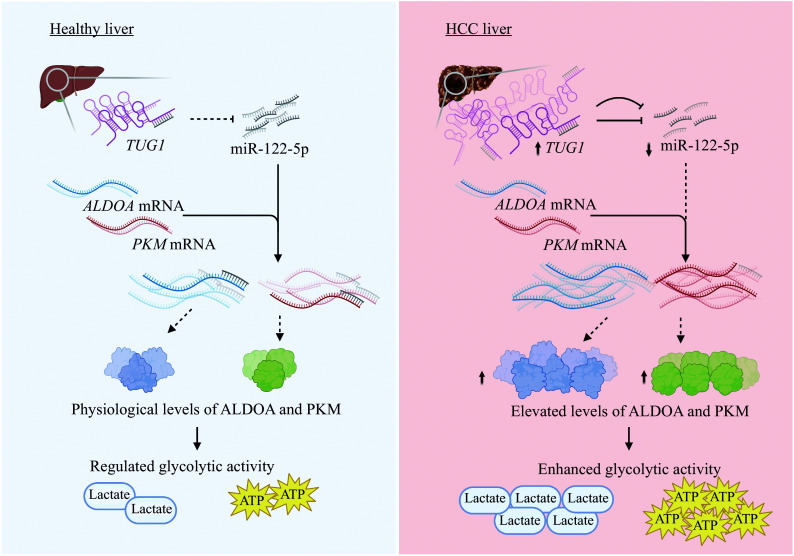
Mechanistic model of glycolysis regulation *via* the *TUG1*/miR-122-5p/ *PKM*/ *ALDOA* axis. *TUG1* is often upregulated in HCC. Then, *TUG1* promotes glycolysis in HCC cells by sponging miR-122-5p, which negatively regulates the expression of glycolysis genes, including *PKM* and *ALDOA*.

To the best of our knowledge, we are the first to examine the effect of lncRNA *TUG1* on the transcriptome of HCC cells and have identified glycolysis as the most significantly affected pathway upon *TUG1* depletion. *TUG1*-knockdown HCC cells exhibited a significant downregulation of several genes encoding glycolytic enzymes. Accordingly, *TUG1* knockdown impaired glucose uptake, ATP production, and lactate formation, reflecting a reduction in glycolytic activity, and ultimately inhibited the proliferation of HCC cells. Clinical data revealed a significant upregulation of *TUG1* and glycolysis genes, and high expression of these genes indicated a poor prognosis in HCC patients. Moreover, we observed a positive correlation between the expression levels of *TUG1* and glycolysis genes. Mechanistically, *TUG1* could directly sequester miR-122-5p, which negatively regulated the expression of glycolysis genes such as *PKM* and *ALDOA*, and the inhibition of miR-122-5p alleviated the suppression of glycolytic activity caused by *TUG1* depletion. Supporting this model, we found a negative correlation between the expression levels of miR-122-5p and glycolysis genes. Collectively, we proposed the *TUG1*/miR-122-5p/ *PKM*/ *ALDOA* axis as a novel regulatory mechanism promoting glycolysis in HCC cells ( ***Fig. 7***).

Despite the debated role of *TUG1* in HCC, most studies, including ours, support the tumor-promoting role of *TUG1* in driving the progression of HCC ^[ 11] ^; while some studies have found *TUG1* to function as a tumor suppressor in other cancers. For example, in glioma, overexpression of *TUG1* could suppress the tumorigenicity of temozolomide resistance by downregulating the expression of EZH2 and promoting apoptosis *via* an intrinsic pathway facilitated by caspase-3 and caspase-9 ^[ 15– 16] ^. These results contradict most of the findings in other types of cancer, suggesting *TUG1* may influence tumorigenesis in a cancer-type-specific manner. In the present study, we identified the antiglycolytic miR-122-5p as the target for *TUG1* sponging. Alternatively, previous studies identified several lncRNAs as upstream regulators for miR-122-5p expression in HCC ^[ 17– 18] ^. Additionally, miR-122-5p has been reported to negatively regulate a wide range of oncogenic genes in HCC, including *TLR4*, *BCL2L2*, and *TGFβ* ^[ 19– 21] ^. Similarly, numerous studies have emphasized the tumor-suppressive role of miR-122-5p in various types of cancer, including gastric, breast, and ovarian cancer ^[ 22] ^. Conversely, miR-122-5p has also been shown to exhibit an oncogenic role in renal cancer ^[ 23] ^. Collectively, mounting evidence supports the tumor-suppressing role of miR-122-5p, which could potentially inhibit the progression of HCC.

Metabolic reprogramming has been considered one of the critical hallmarks of cancer, providing sufficient energy and building blocks to support the uncontrolled proliferation of cancer cells ^[ 14] ^. Aerobic glycolysis, termed the Warburg effect, has been proposed to promote the initiation and progression of HCC ^[ 14] ^. However, current knowledge regarding the underlying mechanism of reprogramming in cancer remains inadequate. The present study showed that depletion of miR-122-5p *via* *TUG1*-mediated sponging increased the expression of several glycolytic enzymes, such as ALDOA and PKM, thereby promoting glucose breakdown in HCC. In line with our findings, knockdown of *TUG1* alleviated the suppression of miR-453-3p and miR-524-5p and inhibited glycolysis in HCC cells ^[ 10, 24] ^. ALDOA (aldolase A), the most abundant isoform in cancer, is one of the key glycolytic enzymes that has been reported to be closely associated with patient prognosis and survival in cancer ^[ 25] ^. Several studies have also identified PKM (pyruvate kinase M, especially the PKM2 isoform) as a crucial oncogene that is upregulated in most types of cancer, including HCC, to promote cancer progression ^[ 26] ^. Some studies have demonstrated the dual roles of ALDOA and PKM2 in metabolism and DNA repair pathways, suggesting that these two enzymes not only promote the progression of cancer through rewiring energy production pathways but also help cancer cells resist apoptosis ^[ 27- 28] ^. Taken together, ALDOA and PKM may be critical targets for a novel anti-tumor approach in combination with *TUG1* silencing to prevent HCC progression.

Many RNAi-based therapeutics have advanced into clinical trials, demonstrating the viability of modulating non-coding RNAs in treating various human cancers and other diseases ^[ 29] ^. Thus, with an unprecedented speed of RNA therapeutic development, we can establish a novel RNAi-based therapy targeting the *TUG1*/miR-122-5p/ALDOA/PKM axis for HCC treatment. Alternatively, therapeutic strategies that directly target tumor glycolysis are attractive areas of cancer research ^[ 30] ^. Preclinical investigations have identified inhibitors of glycolytic enzymes, such as 2-deoxy-D-glucose, that have been demonstrated to render HCC cells susceptible to chemotherapy ^[ 31] ^. Nevertheless, effective methods to specifically target the glycolysis of cancer cells to enhance therapeutic efficacy remain a formidable challenge.

The limitations of the present study include that we only focused on the dominant form of *TUG1* variants in our computational prediction of miRNA binding, as evidence suggests that lncRNAs may undergo splicing to generate multiple isoforms ^[ 32] ^. Therefore, studying other *TUG1* variants might provide additional insight into their molecular function in HCC. Second, the gain-of-function study of *TUG1* could not be completed because of the large size of *TUG1* (approximately 7 kb), which could not be packaged into the lentiviral vector for overexpression ^[ 33] ^. Furthermore, the transfection of plasmids with large inserts lowers DNA uptake by the cells ^[ 34] ^. We attempted to overexpress *TUG1* from native loci by CRISPR activation (CRISPRa), but none of the guide RNAs showed satisfactory results. Nevertheless, transgenic expression of lncRNAs with their endogenous length and secondary structure has been challenging to truly reflect their bona fide functions ^[ 35] ^. It is important to acknowledge the inherent limitations of short-hairpin RNAs targeting *TUG1*, including the potential for off-target effects. These effects may arise from partial sequence homology with unrelated transcripts, potentially influencing the global gene expression profiles observed in our RNA-seq data. Although the GPP Web Portal algorithm from the Broad Institute, used for shRNA design, is known to enhance targeting specificity and minimize off-target effects, the discrepancy in the number of DEGs between sh1- *TUG1* and sh2- *TUG1* suggests that some degree of off-target activity cannot be entirely excluded. We have therefore added a note of caution regarding the interpretation of our transcriptomic data, particularly in relation to the extent of gene expression changes observed.

In conclusion, lncRNA *TUG1* plays a critical role in the progression of cancers, including HCC. Nevertheless, the molecular function of *TUG1* remains not fully elucidated. Through transcriptomic analysis of *TUG1*-knockdown HCC cells and a series of bioinformatic analyses, we revealed that *TUG1* acted as a sponge for miR-122-5p, which negatively regulates the expression of key glycolysis genes, including *PKM* and *ALDOA*. An inverse correlation between the expression levels of miR-122-5p and glycolysis genes in clinical samples further substantiated these results. Together, we propose a novel mechanism through which *TUG1* promotes glycolysis in HCC cells and highlight the therapeutic potential of targeting the *TUG1*/miR-122-5p axis for HCC treatment. We envision that RNA-based therapeutics could one day modulate non-coding RNA activity in cancer cells, potentially halting or reversing HCC progression in patients and transforming current cancer treatment paradigms.

## SUPPLEMENTARY DATA

Supplementary data to this article can be found online.
