# Supplementary material for: Long non-coding RNA TUG1 regulates multiple glycolytic enzymes in hepatocellular carcinoma cells by sponging microRNA-122-5p
Source: J Biomed Res. 2025 Jul 15;39(5):515–29. doi: 10.7555/JBR.39.20250056 (PMC12481678; doi:10.7555/JBR.39.20250056)
Supplement: Supplementary file 1 — Supplementary data to this article can be found online. [file jbr-39-5-515-Supplementary.pdf]

Available online at www.jbr-pub.org.cn

Open Access at PubMed Central

Journal of Biomedical Research, 2025 39(5): 1–3

JBR

Supplementary Data

Long non-coding RNA *TUG1* regulates multiple glycolytic enzymes in hepatocellular carcinoma cells by sponging microRNA-122-5p

Thammachanok Boonto[1,2,3], Chinnatam Phetkong[1,2,4], Chaiyaboot Ariyachet[1,2,✉]

[1]*Department of Biochemistry, Faculty of Medicine, Chulalongkorn University, Bangkok 10330, Thailand;*
[2]*Center of Excellence in Hepatitis and Liver Cancer, Faculty of Medicine, Chulalongkorn University, Bangkok 10330, Thailand;*
[3]*Medical Biochemistry Program, Department of Biochemistry, Faculty of Medicine, Chulalongkorn University, Bangkok 10330, Thailand;*
[4]*Medical Science Program, Faculty of Medicine, Chulalongkorn University, Bangkok 10330, Thailand.*

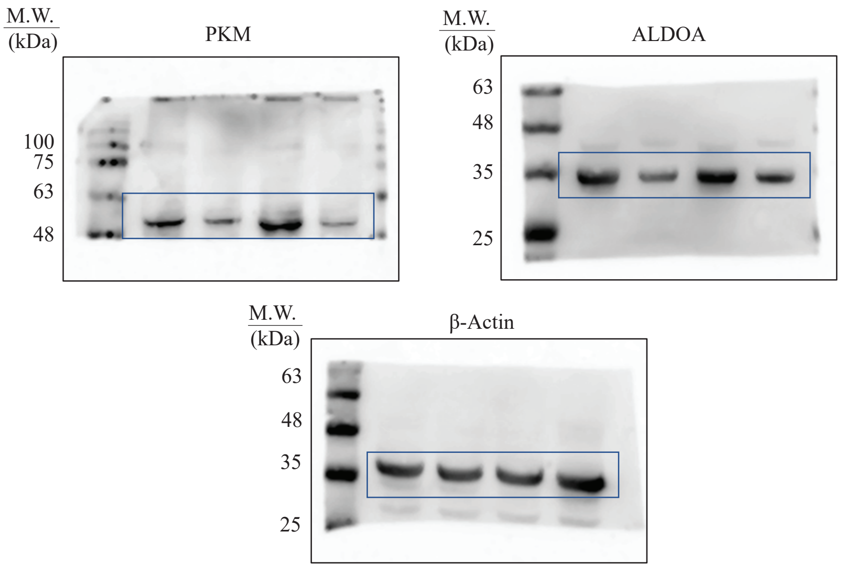

Supplementary Fig. 1 **Uncropped Western blots.**

✉Corresponding author: Chaiyaboot Ariyachet, Department of Biochemistry, Faculty of Medicine, Chulalongkorn University, 1873 Paettayaphat Building, Rama Ⅳ Road, Pathumwan, Bangkok 10330, Thailand. E-mail: chaiyaboot.a@chula.ac.th or cariyach@gmail.com.

Received: 11 February 2025; Revised: 30 June 2025; Accepted: 01 July 2025; Published online: 15 July 2025

CLC number: R735.7, Document code: A

The authors reported no conflict of interests.

This is an open access article under the Creative Commons Attribution (CC BY 4.0) license, which permits others to distribute, remix, adapt and build upon this work, for commercial use, provided the original work is properly cited.

© 2025 by Journal of Biomedical Research.

https://doi.org/10.7555/JBR.39.20250056

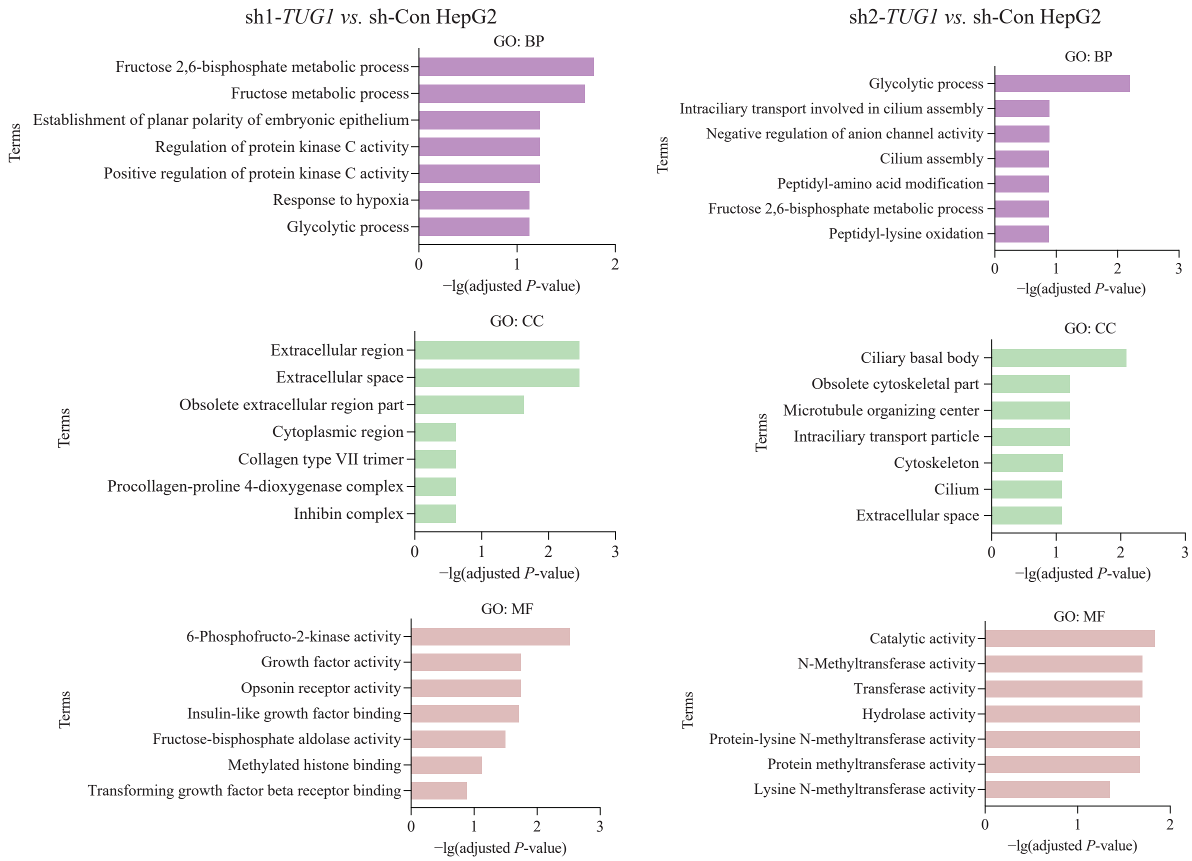

Supplementary Fig. 2 **GO term analysis of downregulated DEGs in *TUG1*-knockdown HepG2 cells.** GO term analysis from RNA-seq data (GSE273253) of control (sh-Con) and *TUG1*-knockdown (sh1- and sh2-*TUG1*) HepG2 cells. Abbreviation: GO, gene ontology; DEG, differentially expressed gene; BP, biological process; CC, cellular components; MF, molecular function.

A

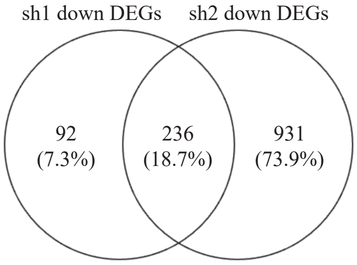

B

Pathway	Fold enrichment	FDR
HIF-1 signaling pathway	8.885 393 913	0.000 585
Fructose and mannose metabolism	18.342 953 34	0.000 602 555

C

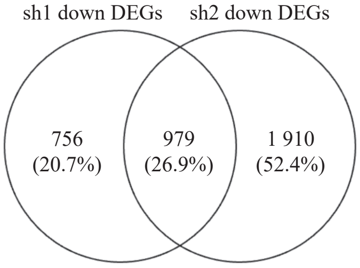

D

Pathway	Fold enrichment	FDR
Metabolic pathways	1.802 776	5.60×10^{-7}
HIF-1 signaling pathway	4.041 445	0.000 143
Fructose and mannose metabolism	7.067 127	0.000 312
Glycolysis/Gluconeogenesis	4.641 098	0.000 528
Biosynthesis of nucleotide sugars	6.303 113	0.000 528

Supplementary Fig. 3 **KEGG pathway analysis of commonly downregulated DEGs following *TUG1* knockdown in HepG2 cells.** A: Venn diagram illustrating the overlap of DEGs between *TUG1*-knockdown (sh1- and sh2-*TUG1*) and control (sh-Con) HepG2 cells. Genes were considered downregulated if their |fold change (FC)| was greater than 1.5 and the *P*-value was less than 0.05. B: KEGG pathway enrichment analysis of the overlapping downregulated DEGs identified in panel (A) (|FC| > 1.5, P < 0.05). C: Venn diagram showing the overlap of downregulated DEGs between *TUG1*-knockdown and control HepG2 cells, using a less stringent threshold of |FC| > 1.0 and P < 0.05. D: KEGG pathway enrichment analysis of the overlapping downregulated DEGs identified in panel (C) (|FC| > 1.0, P < 0.05). Abbreviation: DEG, differentially expressed gene; KEGG, Kyoto Encyclopedia of Genes and Genomes; BP, biological process; CC, cellular components; MF, molecular function.

Supplementary Tables

Supplementary Table 1 (available online): Primers used in this study.

Supplementary Table 2 (available online): GSEA analysis of downregulated gene sets upon *TUG1* knockdown in HepG2 cells by Hallmark, WikiPathway, and KEGG medicus.

Supplementary Table 3 (available online): Analysis for correlation of *TUG1* expression with glycolysis candidate genes from TCGA, Starbase v.2/ENCORI, and GEPIA2 databases.

Supplementary Table 4 (available online): Prediction of microRNAs that potentially interact with *TUG1*-regulated glycolysis genes by the multiMiR database.

Supplementary Table 5 (available online): Hybridization patterns of the top five candidate miRNAs with *TUG1* by RNA22 v2 prediction.

Supplementary Table 6 (available online): Analysis for correlation of candidate miRNA expression with glycolysis candidate genes by starBase v2.0.

Supplementary Table 7 (available online): Top five hybridization patterns of miR-122-5p with 3′ UTR of *PKM* and *ALDOA* transcripts predicted by the RNAhybrid.
